# An Overview of MicroRNAs as Biomarkers of ALS

**DOI:** 10.3389/fneur.2019.00186

**Published:** 2019-03-07

**Authors:** Greig Joilin, P. Nigel Leigh, Sarah F. Newbury, Majid Hafezparast

**Affiliations:** ^1^School of Life Sciences, University of Sussex, Brighton, United Kingdom; ^2^Brighton and Sussex Medical School, University of Sussex, Brighton, United Kingdom

**Keywords:** amyotrophic lateral sclerosis, motor neuron disease, biomarkers, non-coding RNA, microRNA

## Abstract

Amyotrophic lateral sclerosis (ALS; MND, motor neuron disease) is a debilitating neurodegenerative disease affecting 4.5 per 100,000 people per year around the world. There is currently no cure for this disease, and its causes are relatively unknown. Diagnosis is based on a battery of clinical tests up to a year after symptom onset, with no robust markers of diagnosis or disease progression currently identified. A major thrust of current research is to identify potential non-invasive markers (“biomarkers”) in body fluids such as blood and/or cerebrospinal fluid (CSF) to use for diagnostic or prognostic purposes. Non-coding RNAs (ncRNAs), including microRNAs (miRNAs), are found at detectable and stable levels in blood and other bodily fluids. Specific ncRNAs can vary in levels between ALS patients and non-ALS controls without the disease. In this review, we will provide an overview of early findings, demonstrate the potential of this new class as biomarkers, and discuss future challenges and opportunities taking this forward to help patients with ALS.

## Introduction

Amyotrophic lateral sclerosis (ALS) is the most prevalent adult onset form of motor neuron disease. As a result of progressive death of motor neurons in the primary motor cortex, brainstem and spinal cord, there is atrophy of the muscles that are innervated by these neurons. This results in muscle weakness and paralysis with death usually occurring within 3–5 years. Over the last decade, significant progress has been made in identifying the genes responsible for familial cases of ALS (fALS). Of these, the most frequently mutated genes are chromosome 9 open reading frame 72 (*C9orf72*), superoxide dismutase 1 (*SOD1*), TAR DNA-binding protein 43 (*TARDBP*; TDP-43), and FUS RNA binding protein (*FUS*), accounting for over 70% of fALS cases (1). Nevertheless, fALS constitutes approximately 10% of all cases, with the genetic underpinnings of sporadic ALS (sALS) mostly unknown, though *C9orf72* is known to account for 5% of sALS cases.

The lack of a common cause has resulted in difficulties not only in timely disease diagnosis resulting in delay of treatment, but in developing drugs and treatments for the disease. Thus, identifying useful biomarkers as tools for early diagnosis, for determining subgroups in relation to pathogenesis and/or phenotype, and as indicators of treatment response, are urgently required. Development of biomarkers that are minimally invasive to obtain, simple to undertake, and time efficient are key and those derived from biofluids, such as blood, are well suited for this. Further, it is not necessary for the biomarkers to underlie the pathology of the disease if it correlates strongly and specifically to the disease. Indeed, this is more difficult to assess in diseases such as ALS where the underlying molecular causes of pathology is unknown or unclear.

One class of molecules increasingly investigated as potential biomarkers are short ncRNA species (those under 100 nucleotides long), which include tRNA, rRNA, piwi-RNA (piRNA), and microRNA (miRNA). MiRNA have been the main focus of most studies to date, driven by a good understanding of their biogenesis and function, an ease in profiling their expression with a range of techniques including microarray, RNA-seq, and RT-qPCR, a relatively simple structure, increased stability from RNase degradation and freeze-thaw cycles, and a presence in a range of biofluids including blood, cerebrospinal fluid (CSF), and urine (2, 3). To date, a number of studies have shown that miRNAs are differentially expressed in ALS patients when compared to controls in a variety of biofluids, including CSF, and in the blood-derived components plasma and serum (4–18) (summarized in Table 1). This review will aim to present recent work identifying miRNA-based biomarkers in biofluids, the possibility of using other ncRNA as biomarkers, and the next steps required to move this into a clinical setting.

**Table 1:** Circulating miRNA-based biomarkers found to be differentially expressed in biofluids.

	**Authors**	**ALS type**	***n***	**Validated changes**		**Controls**	**RNA extraction**	**Profiling technique**	**RT-qPCR validation**	**RT-qPCR Normalization**
				**Increase**	**Decrease**					
Serum	Freischmidt et al. (4)	Sporadic	22	–	MIR132-5p MIR132-3p **MIR143-5p** MIR143-3p LET7B-5p	Age-matched healthy controls	miRNeasy Mini	–	Ncode VILO EXPRESS SYBR GreenER	Spiked in cel-MIR39-3p
	De Felice et al. (5)	Sporadic	72	MIR338-3p	–	Age-matched healthy controls	Trizol	–	miScript RT-qPCR	LET7A
	Freischmidt et al. (6)	Familial	22	–	MIR1825 MIR1915-3p MIR3665 MIR4530 MIR4745-5p	Age-matched healthy controls	QIAzol and miRNeasy Mini	Affymetrix GeneChip 3.0 Array	miScript RT-qPCR	Spiked in cel-MIR39-3p
		Sporadic	14	–	MIR3665 MIR4530 MIR4745-5p					
	Toivonen et al. (7)	–	12	**MIR106B** MIR206	–	Age-matched healthy controls	Norgen Total RNA	Affymetrix GeneChip 2.0 Array	TaqMan miRNA RT-qPCR	Spiked in cel-MIR39-3p
	Freischmidt et al. (8)	Sporadic	18	–	MIR1234-3p MIR1825	Age-matched healthy controls/ Alzheimer's/ Huntington's	QIAzol and miRNeasy Mini	Affymetrix GeneChip 3.0 Array	miScript RT-qPCR	Spiked in cel-MIR39-3p
	Waller et al. (13)	Sporadic	50	**MIR143-3p** MIR206	MIR374B-5p	Age-matched healthy controls/ disease mimics	Norgen Circulating Nucleic Acid Isolation	TaqMan Low Density RT-qPCR arrays	miScript RT-qPCR	MIR17-5p MIR24 MIR223-3p
	Matamala et al. (16)	Sporadic	20	MIR142-3p	MIR1249-3p	Age-matched healthy controls	Trizol LS and miRNeasy Serum/ Plasma	Illumina TruSeq Small RNA on Illumina MiSeq	TaqMan miRNA RT-qPCR	Spiked in cel-MIR39-3p
	Raheja et al. (17)	Sporadic/ Familial	23	Screen only	Screen only	Healthy controls	miRcury	miRNA LNA RT-qPCR arrays	–	–
	Xu et al. (18)	–	10	–	MIR27A-3p	Healthy controls	Trizol or miRNeasy Micro	–	miDETECT A Track miRNA RT-qPCR or TaqMan miRNA RT-qPCR	MIR16-5p
Plasma	Takahashi et al. (9)	Sporadic	48	MIR4649-5p	MIR4299	Age-matched healthy controls	miRNeasy Serum/ Plasma	3D-Gene Human miRNA oligo chip	miScript RT-qPCR	MIR4516
	de Andrade et al. (11)	Sporadic	39	MIR424 MIR206	–	Aged match healthy control	miRVana PARIS	Affymetrix GeneChip array (on muscle)	TaqMan miRNA RT-qPCR	MIR16-5p
	Sheinerman et al. (12)	–	50	MIR206/MIR338-3p MIR9/MIR129-3p MIR335-5p/MIR338-3p		Age-matched healthy controls	Trizol and Ambion Glass fiber Columns	Literature search	TaqMan miRNA RT–qPCR	–
Cerebrospinal Fluid	Freischmidt et al. (4)	Sporadic	22	**MIR143-5p** MIR574-5p	MIR132-5p MIR132-3p **MIR143-3p**	Age-matched healthy controls	miRNeasy Mini	–	Ncode VILO EXPRESS SYBR GreenER	Spiked in cel-MIR39-3p
	De Felice et al. (5)	Sporadic	72	MIR338-3p	–	Age-matched healthy controls	Trizol	–	miScript RT-qPCR	MIR24
	Benigni et al. (10)	Sporadic	24	MIR181A-5p	LET7A-5p LET7B-5p LET7F-5p MIR15b-5p MIR21-5p MIR195-5p MIR148A-3p	Age-matched healthy controls	miRNeasy Mini	Human miFinder 384HC miRNA PCR array	SYBR Green RT-qPCR	Spiked in cel-MIR39-3p MIR608 MIR328-3p
	Waller et al. (14)	Sporadic	32	Screen only	Screen only	Age-matched healthy controls/disease mimics	miRVana PARIS	Illumina TruSeq Small RNA on Illumina HiScanSq	miScript II RT-qPCR	Spiked in cel-MIR39-3p MIR30A-5p
Whole Blood	Liguori et al. (15)	Sporadic	56	-	LET7A-5p LET7D-5p LET7F-5p LET7G-5p LET7I-5p MIR15A-5p MIR15B-5p MIR151A-5p MIR151B MIR16-5p MIR22-3p MIR23A-3p MIR26A-5p MIR26B-5p MIR27B-3p MIR28-3p MIR30B-5p MIR30C-5p MIR93-5p MIR103A-3p **MIR106B-3p** MIR128-3p MIR130A-3p MIR130B-3p MIR144-5p MIR148A-3p MIR148B-3p MIR182-5p MIR183-5p MIR186-5p MIR221-3p MIR223-3p MIR342-3p MIR425-5p MIR451A MIR532-5p MIR550A-3p MIR584-5p	Age-matched healthy controls	PAXgene Blood RNA	Illumina TruSeq Small RNA on Illumina HiSeq2500	TaqMan Advanced miRNA RT-qPCR	MIR484

*Those miRNA underlined show consistent directional changes between control and ALS cases while those in bold show contrasting directional changes between control and ALS cases*.

## Existing Circulating RNA Biomarkers for ALS

### Serum-Based Biomarkers

Freischmidt and colleagues have undertaken a number of studies to identify potential miRNA-based biomarkers in the ALS patient serum (4, 6, 8). Their first study selected ten miRNAs previously identified to regulate the ALS-related gene *TARDPB* and found five miRNAs were differentially expressed in serum of sALS patients (4). Their later study focused on miRNA expression in serum from fALS patients using Affymetrix miRNA array chips, and found downregulation of a set of 30 potential miRNA biomarkers for the disease [Table 1; 3]. Four miRNA were selected based on their false discovery rate (FDR)-adjusted *p*-value (MIR1915-3p, MIR3665, MIR4530, MIR4745-5p) and their downregulation validated with RT-qPCR in the fALS patients, and all but MIR1915-3p were further observed to be downregulated in sALS patients. While increased variability was observed in sALS patients, this suggested that there may be similarities in the miRNA profile between the two groups. Curiously, these three miRNAs (MIR3665, MIR4530, MIR4745-5p) were found not to be differentially expressed in their most recent study using sALS patients, which described only MIR1234-3p and MIR1825 as being downregulated (8). An interesting aspect of their 2014 study was investigating miRNA expression in non-symptomatic patients who had ALS-related genetic mutations, but predicted to present disease symptoms within the next 20 years. These predicted pre-symptomatic carriers shared 91.7% of the downregulated miRNA of symptomatic patients, although to a lesser dysregulation. This suggests that these biomarkers may be present before symptoms and could be used to identify potential ALS cases. Furthermore, considering there were differences between pre-symptomatic and symptomatic patients in the degree of dysregulation, this may suggest that these biomarkers could change with time. However, further work would be needed to determine this and if it would apply to sALS cases along with whether these biomarkers are specific to ALS itself.

Other studies have also identified potential biomarkers that may be differentially expressed in serum from ALS patients. The upregulation of MIR143-3p and MIR206, and the downregulation of MIR374B-5p were observed in 23 sALS patients and were further validated in an additional 27 sALS patients (13). Of these, 22 samples were in a longitudinal study and MIR143-3p and MIR374B-5p both became more dysregulated, suggesting a link to disease progression, though MIR206 remained stable for at least 3 months later. Another study using patient serum also found MIR206 upregulation in ALS patients along with MIR106b, differences that were reflected in a SOD1-G93A mouse model of ALS (7). MIR206, described as a myoMiR due to its high abundance in skeletal muscle tissue, is one of the few miRNA biomarkers identified across multiple studies, including those described below in serum and plasma (11, 12). The working hypothesis has been that as a result of muscle death, MIR206 is released from the muscle fibers and into the blood stream as a waste product (19). However, MIR206 has been identified as a blood-based biomarker for other muscle-related diseases and therefore not specific to ALS (20, 21). Nonetheless, it could play an important role in helping to identify ALS patients if used in conjunction with other biomarkers to help distinguish from ALS-like conditions. Lastly, one study has investigated the exosomes present in serum, and investigated a single miRNA (MIR27A-3p) based on the research group's previous work with myoblast exosomes (18). However, the normalization to MIR16-5p may limit the interpretation of this data as it has been shown to be dysregulated in ALS (15, 22) and no evidence was shown that MIR16-5p was stable. Nonetheless, with a fuller investigation, identifying dysregulated miRNA present in exosomes in ALS may provide clues as to the source, destination, and thus function of circulating miRNA in ALS.

### Plasma-Based Biomarkers

Two studies have investigated biomarkers in sALS patients using plasma; the portion of blood which contains clotting factors. Using microarray analysis followed by RT-qPCR, Takahashi et al. (9) found significant upregulation of MIR4649-5p and downregulation of MIR4299 in ALS patients compared to healthy controls. Interestingly, this study incorporated a follow up analysis of the expression of miRNAs in seven of the patients, including one patient 24 months later. However, no significant change in the expression of any of the miRNAs were found, although there was a trend for an increase of MIR663b over time. Similarly, in another study, while MIR424 and MIR206 were found to be overexpressed in plasma of sALS patients, they did not show significant changes over 6 and 12 months in a cohort of sALS patients (11). This lack of change in MIR206 over time is consistent with the above results of Waller et al. (13). This suggests that for these miRNA, their expression levels are not correlated with disease progression and changes in the patient condition. This may mean that they may only be suitable as diagnostic markers and not useful in tracking treatment responses in disease.

### Cerebrospinal Fluid-Based Biomarkers

In addition to serum and plasma, differential expression of ncRNA has also been investigated in CSF. Although CSF is not as easily obtainable as blood, changes in expression may potentially be more insightful due to its close proximity to the central nervous system. Using RT-qPCR, De Felice et al. (5) not only found MIR338-3p to be over-expressed in serum, but also in CSF, blood leukocytes, and spinal cord tissue in ALS patients compared to controls and other patient groups (including patients with Alzheimer's and Parkinson's disease). *In situ* hybridization staining of spinal cord tissue post mortem found that MIR338-3p was localized in the dorsal root gray matter and overexpressed in ALS patients, suggesting a potential source of the miRNA. In contrast, Freischmidt et al. (4) used the biomarkers identified in their serum work to find out if there were similar changes in the CSF. While four of those miRNAs were dysregulated, only MIR143-3p showed a significant correlative relationship between the serum and CSF, suggesting there is low correlation in miRNA expression between these two biofluids. Combined with generally higher concentrations of miRNA in the serum, the authors concluded that there might be separate regulatory mechanisms underlying the levels of miRNAs in these two body compartments. This is supported by other papers looking into CSF which have shown very little overlap with other serum studies, but studies that have looked at both within the same sample groups are limited.

## Emerging Themes in ALS Biomarker Discovery

Recently, two main themes are starting to emerge in biomarker discovery, including in those for ALS. Firstly, it is becoming evident that seeking to identify singular biomarkers for disease is unlikely, underscored by the minimal overlap demonstrated by the above studies. In a study to identify miRNA biomarkers in CSF, using ratios between the expression of two miRNA as determined by RT-qPCR increased sensitivity and specificity in identifying sALS cases compared to using a single miRNA (10). The study pointed out that the use of more than one miRNA as a “biomarker signature” is preferable as it reduces the dependency on variation between individuals. The pairing of the upregulated MIR181A-5p, with either of their two downregulated miRNA, MIR21-5p and MIR15B-5p, increased both the sensitivity and the specificity, with MIR15B-5p increased by 15% on average. Another study has also used this concept for miRNA present in serum, using a number of pairs to identify not only patients with ALS, but other neurological disorders such as Alzheimer's disease, frontotemporal dementia, and Parkinson's disease (12). Having identified 37 brain- or inflammation-enriched miRNA, they found the combination of the three pairs of miRNAs (MIR206/MIR338-3p, MIR9/MIR129-3p, and MIR335-5p/MIR338-3p) were able to clearly distinguish between ALS and control patients in their cohort with a sensitivity of 84% and a specificity of 82%. Furthermore, other paired combinations were able to differentiate between other neurodegenerative diseases and ALS. Sheinerman et al. (12) found an 8-fold increase in MIR206 levels in the plasma of ALS patients when compared to the controls and this was enough to distinguish ALS patients from controls by itself. Therefore, on the whole, pairs of miRNA were able to distinguish between the various diseases and controls with higher accuracy than could be achieved by an individual miRNA.

Secondly, recent advances have improved the generation of high quality libraries from small amounts of starting RNA, allowing unbiased screening of potential ncRNA biomarkers by the RNA-seq technique. In one of the first studies, following on from their work with serum, Waller et al. (14) used RNA-seq to profile miRNA expression in the CSF of ALS patients. While they were able to successfully sequence the miRNA and identify potential candidates, they were unable to confirm those with RT-qPCR because of technical issues. Nonetheless, it supports the conclusion of the above studies that differences in miRNA can be detected in CSF and that CSF could be a source of biomarkers.

More recently, one study has used total blood to screen for miRNA biomarkers in ALS using RNA-seq (15). Following identification of 42 differentially expressed miRNA in the discovery cohort, 38 were validated using RT-qPCR, most of which have been previously reported in other papers. Interestingly, seven of the miRNAs (MIR30B-5p, MIR30C-5p, MIR106B-3p, MIR128-3p, MIR148B-3p, MIR186-5p, MIR342-3p) were able to distinguish between spinal and bulbar onset, with decreased expression present for those with spinal onset. Furthermore, this study also carried out RNA-seq on mRNAs in the same samples to help identify targets that could be regulated by the miRNA. The use of total blood, however, limits the interpretation of these results due to the presence of red blood cells in the samples and the possibility of variable numbers of different types of white blood cells between patient and control groups.

Matamala et al. (16) also utilized RNA-seq for the identification of ALS biomarkers, but started by profiling serum samples from transgenic mouse models of ALS, followed by RT-qPCR validation in human samples. While a number of miRNAs were found to change in levels between the ALS model and controls, there was limited cross-validation when this was taken forward to the human studies. Two miRNA that did show differences between ALS patients and controls in the human studies were MIR142-3p and MIR1249-3p. The authors found that MIR142-3p seemed to correlate negatively with a decline in clinical disability scale ALSFRS-R in patients, thus suggesting that this could be used to measure the effect of any disease-slowing treatment. Further, it was found to potentially target the expression of the ALS genes *TARDBP* and *C9orf72*. Interestingly, Matamala et al. (16) also briefly described the detection of non-miRNA ncRNA with their RNA-seq, but did not state if they were differentially expressed or whether they were investigated further. As such, there may be a range of potential biomarkers that have not yet been identified. Indeed, several other ncRNA species have been detected in serum including rRNA and tRNA (23–25). These have also been highlighted as potential biomarkers in diseases other than ALS in blood (26–29) and other tissues (24, 30–32). To this end, we are currently using RNA-seq to identify potential biomarkers in ALS within the full cohort of ncRNA species, and early results suggest that we have potential candidates, which include miRNA, piRNA, and tRNA.

## Challenges and Opportunities

Across these studies, there is very little overlap in the miRNA species as potential biomarkers in the biofluids (see Table 1), and there are multiple potential reasons for this. Firstly, as these are mostly from elderly human patients, some of these patients could have other conditions which could alter the miRNA composition of the biofluids themselves, thus confound the detection of ALS-specific biomarkers; careful screening of patients therefore is required. Further, some of these studies do not include patients from ALS disease mimics to help identify ALS-specific markers. This is important as some biomarkers identified such as MIR206 are not specific to ALS as described above. Additionally, most of these studies have been carried out on samples from one population group. As differences may exist between different populations with the disease, the lack of cross-validation of changes in miRNA expression between studies may be reflective of differences in the patient population, whether that be mediated genetically and/or environmentally. The number of patients also differ, from 12 to 72 ALS patients, and so the statistical power for some of these biomarkers is limited.

**Figure 1:**
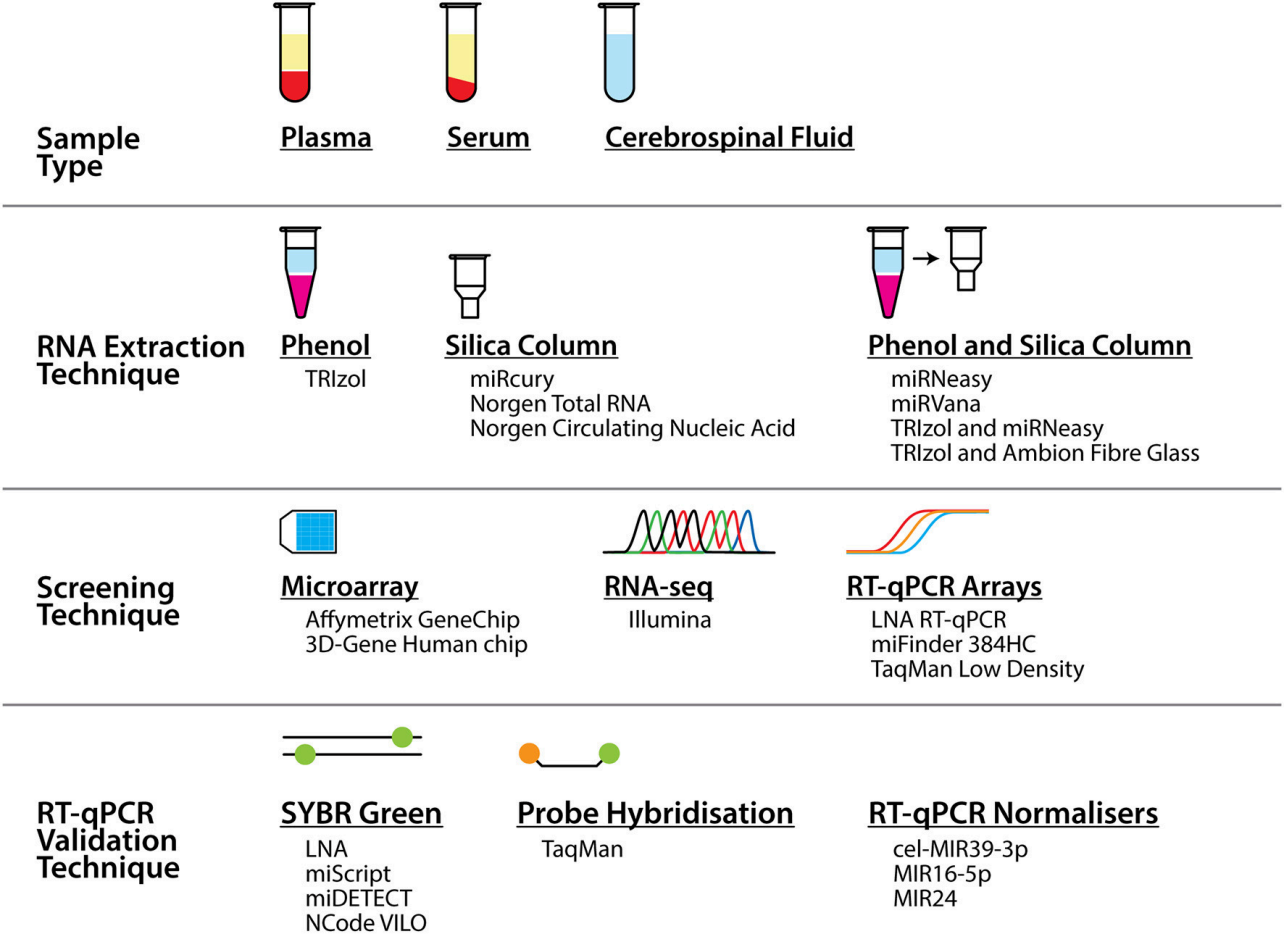
Summary of the different sample types, techniques, and tools that were used to profile miRNA biomarkers in ALS in the studies presented in this review. As can be seen alongside Table 1, numerous different combinations of these sample types and tools across the techniques were employed across a number of studies.

Alternatively, the causes could be related to the methodology of the study, from the extraction of the biofluids and RNA, through to the screening and validation of the miRNA biomarkers. As seen in Table 1 and Figure 1, a range of different workflows have been undertaken across all the studies, all of which may contribute to differences in the changes that are detected. In addition, some of these factors potentially could affect the strength of some of these studies. For example, how the samples were collected and processed may vary. Some of the studies did not describe their collection procedures, and it is well known that differences in the centrifugation time post-collection, speed of centrifugation, and temperature can all affect the quality and quantity of RNA in the samples (33, 34).

Further, techniques used to normalize the RT-qPCR could be considered questionable in a number of studies. Due to the minimal and varied amount of RNA in biofluids, and the resultant difficulty in quantifying the RNA concentration, most miRNA RT-qPCR kits used fixed sample volumes instead of fixed total RNA amounts. Therefore, miRNA RT-qPCR normalization must control for input RNA, not just for technical variation, by crucially using a reliable target as a normalizer. However, no universal normalizer for biomarker work exists, and identification of a suitable normalizer is a problem across all biofluid biomarker studies (35, 36). Normalizers should be selected per study from those ncRNA with the most stable expression in their screen and then validated. Instead, a number of the above studies have used the synthetic spike-in cel-MIR39B, but this would only control for technical variation introduced from RNA extraction onwards, not for the total RNA amount in the starting volume. Others have used miRNA recommended as normalizers such as MIR16-5p, but as described above, it has been shown to be regulated in ALS (15, 22), and also in stress and in red blood cells (37, 38). Indeed, as some studies did not check for hemolysis in their samples, the observed changes may be due to released miRNA from the lysed red blood cells. Taken together, this underlies why there may be limited cross-validation between studies and thus careful consideration of identifying objective normalizers are required.

One question is how do these miRNA-based biomarkers compare to other biomarkers for ALS? While the properties of miRNA as biomarkers as described above are ideal, there are other molecules such as DNA, RNA, protein, and metabolites that could also be used. One of the most commonly used biomarkers in ALS is the neurofilament proteins, which form part of the cytoskeleton of neurons and has been found to be present in both CSF and serum. Studies have shown that neurofilaments are able to help with identification of ALS cases but like MIR206 are not specific to ALS, and rather a measure of axonal death. As such, it is likely that singular miRNA or neurofilaments by themselves will not be able to help with ALS diagnosis or prognosis, but they could form part of any potential biomarker signature. Therefore, it is likely that an integrative approach is required, using data on the levels of a number of ncRNA biomarkers, as has been shown for other diseases (39). Such approaches include utilizing multiple biomarkers, including both miRNA and non-miRNA based biomarkers, and integrating them into a signature model such as a discriminant model, or by using ratios of miRNA expression and using them to help with classification of the disease state, and a number of the above studies have done this. Together, this may help allow ALS patients to be specifically identified, not only from healthy controls but from disease mimics. Therefore, taking this work forward into larger cohorts of patients is vital to test integrating these biomarkers together.

Indeed, opportunities from well-designed studies to validate their biomarkers in separate and larger cohorts could allow for these biomarkers to be used clinically. Further, these studies have been designed first and foremost to find biomarkers for ALS with little attempt to determine the biology underlying these changes, as presence alone does not infer function. Nonetheless, considering the wide and varied biological roles of miRNAs, determining their biological function will be important. Future studies need to include their source and destination, potentially by investigating exosomes and their contents and function. These studies would provide new insights into the mechanisms that may underlie ALS. Therefore, not only do larger cohorts need to be screened but proper experimental design needs to be undertaken to ensure that results are valid and can be used to progress the field further.

What is ultimately being sought is a set of biomarkers that are able to help with the diagnosis and prognosis of ALS patients. Diagnosis and prognosis of patients based on an ncRNA biomarker could assist with the development of tailored and targeted treatments to extend or improve patients' quality of life. As such, these studies have shown that there is potential here for ncRNA-based biomarkers to be identified, and with careful consideration, future work will help to further refine this to progress this to the clinical setting.

## Author Contributions

GJ wrote and edited the manuscript. MH, PL, and SN critically reviewed and edited the manuscript.

### Conflict of Interest Statement

The authors declare that the research was conducted in the absence of any commercial or financial relationships that could be construed as a potential conflict of interest.
