# Corrigendum: An Overview of MicroRNAs as Biomarkers of ALS

**DOI:** 10.3389/fneur.2019.01129

**Published:** 2019-10-23

**Authors:** Greig Joilin, P. Nigel Leigh, Sarah F. Newbury, Majid Hafezparast

**Affiliations:** ^1^School of Life Sciences, University of Sussex, Brighton, United Kingdom; ^2^Brighton and Sussex Medical School, University of Sussex, Brighton, United Kingdom

**Keywords:** amyotrophic lateral sclerosis, motor neuron disease, biomarkers, non-coding RNA, microRNA

In the original article, there was a mistake in Table 1 as published. Some of the miRNAs listed in the table were incorrectly placed in the wrong column and/or row. The corrected Table 1 appears below.

**Table 1:** Circulating miRNA-based biomarkers found to be differentially expressed in biofluids.

	**Authors**	**ALS type**	***n***	**Validated changes**		**Controls**	**RNA extraction**	**Profiling technique**	**RT-qPCR validation**	**RT-qPCR Normalization**
				**Increase**	**Decrease**					
Serum	Freischmidt et al. (4)	Sporadic	22	–	MIR132-5p MIR132-3p **MIR143-5p** MIR143-3p LET7B-5p	Age-matched healthy controls	miRNeasy Mini	–	Ncode VILO EXPRESS SYBR GreenER	Spiked in cel-MIR39-3p
	De Felice et al. (5)	Sporadic	72	MIR338-3p	–	Age-matched healthy controls	Trizol	–	miScript RT-qPCR	LET7A
	Freischmidt et al. (6)	Familial	22	–	MIR1825 MIR1915-3p MIR3665 MIR4530 MIR4745-5p	Age-matched healthy controls	QIAzol and miRNeasy Mini	Affymetrix GeneChip 3.0 Array	miScript RT-qPCR	Spiked in cel-MIR39-3p
		Sporadic	14	–	MIR3665 MIR4530 MIR4745-5p					
	Toivonen et al. (7)	–	12	**MIR106B** MIR206	–	Age-matched healthy controls	Norgen Total RNA	Affymetrix GeneChip 2.0 Array	TaqMan miRNA RT-qPCR	Spiked in cel-MIR39-3p
	Freischmidt et al. (8)	Sporadic	18	–	MIR1234-3p MIR1825	Age-matched healthy controls/ Alzheimer's/ Huntington's	QIAzol and miRNeasy Mini	Affymetrix GeneChip 3.0 Array	miScript RT-qPCR	Spiked in cel-MIR39-3p
	Waller et al. (13)	Sporadic	50	**MIR143-3p** MIR206	MIR374B-5p	Age-matched healthy controls/ disease mimics	Norgen Circulating Nucleic Acid Isolation	TaqMan Low Density RT-qPCR arrays	miScript RT-qPCR	MIR17-5p MIR24 MIR223-3p
	Matamala et al. (16)	Sporadic	20	MIR142-3p	MIR1249-3p	Age-matched healthy controls	Trizol LS and miRNeasy Serum/ Plasma	Illumina TruSeq Small RNA on Illumina MiSeq	TaqMan miRNA RT-qPCR	Spiked in cel-MIR39-3p
	Raheja et al. (17)	Sporadic/ Familial	23	Screen only	Screen only	Healthy controls	miRcury	miRNA LNA RT-qPCR arrays	–	–
	Xu et al. (18)	–	10	–	MIR27A-3p	Healthy controls	Trizol or miRNeasy Micro	–	miDETECT A Track miRNA RT-qPCR or TaqMan miRNA RT-qPCR	MIR16-5p
Plasma	Takahashi et al. (9)	Sporadic	48	MIR4649-5p	MIR4299	Age-matched healthy controls	miRNeasy Serum/ Plasma	3D-Gene Human miRNA oligo chip	miScript RT-qPCR	MIR4516
	de Andrade et al. (11)	Sporadic	39	MIR424 MIR206	–	Aged match healthy control	miRVana PARIS	Affymetrix GeneChip array (on muscle)	TaqMan miRNA RT-qPCR	MIR16-5p
	Sheinerman et al. (12)	–	50	MIR206/MIR338-3p MIR9/MIR129-3p MIR335-5p/MIR338-3p		Age-matched healthy controls	Trizol and Ambion Glass fiber Columns	Literature search	TaqMan miRNA RT–qPCR	–
Cerebrospinal Fluid	Freischmidt et al. (4)	Sporadic	22	**MIR143-5p** MIR574-5p	MIR132-5p MIR132-3p **MIR143-3p**	Age-matched healthy controls	miRNeasy Mini	–	Ncode VILO EXPRESS SYBR GreenER	Spiked in cel-MIR39-3p
	De Felice et al. (5)	Sporadic	72	MIR338-3p	–	Age-matched healthy controls	Trizol	–	miScript RT-qPCR	MIR24
	Benigni et al. (10)	Sporadic	24	MIR181A-5p	LET7A-5p LET7B-5p LET7F-5p MIR15b-5p MIR21-5p MIR195-5p MIR148A-3p	Age-matched healthy controls	miRNeasy Mini	Human miFinder 384HC miRNA PCR array	SYBR Green RT-qPCR	Spiked in cel-MIR39-3p MIR608 MIR328-3p
	Waller et al. (14)	Sporadic	32	Screen only	Screen only	Age-matched healthy controls/disease mimics	miRVana PARIS	Illumina TruSeq Small RNA on Illumina HiScanSq	miScript II RT-qPCR	Spiked in cel-MIR39-3p MIR30A-5p
Whole Blood	Liguori et al. (15)	Sporadic	56	-	LET7A-5p LET7D-5p LET7F-5p LET7G-5p LET7I-5p MIR15A-5p MIR15B-5p MIR151A-5p MIR151B MIR16-5p MIR22-3p MIR23A-3p MIR26A-5p MIR26B-5p MIR27B-3p MIR28-3p MIR30B-5p MIR30C-5p MIR93-5p MIR103A-3p **MIR106B-3p** MIR128-3p MIR130A-3p MIR130B-3p MIR144-5p MIR148A-3p MIR148B-3p MIR182-5p MIR183-5p MIR186-5p MIR221-3p MIR223-3p MIR342-3p MIR425-5p MIR451A MIR532-5p MIR550A-3p MIR584-5p	Age-matched healthy controls	PAXgene Blood RNA	Illumina TruSeq Small RNA on Illumina HiSeq2500	TaqMan Advanced miRNA RT-qPCR	MIR484

*Those miRNA underlined show consistent directional changes between control and ALS cases while those in bold show contrasting directional changes between control and ALS cases*.

The authors apologize for this error and state that this does not change the scientific conclusions of the article in any way. The original article has been updated.

